# Effectiveness of design thinking pedagogy in SCCT-based career guidance: Students’ career decision-making self-efficacy and perceived value

**DOI:** 10.1371/journal.pone.0348205

**Published:** 2026-05-05

**Authors:** Yahong Cai, Nalini Arumugam, Yukai Chen

**Affiliations:** 1 School of Education Sciences, Hanshan Normal University, Chaozhou, Guangdong, China; 2 Faculty of Education, Languages, Psychology, and Music (FoELPM), SEGi University, Petaling Jaya, Malaysia; 3 School of Geography, Tourism and Teochew Cuisine, Hanshan Normal University, Chaozhou, Guangdong, China; The John Paul II Catholic University of Lublin, POLAND

## Abstract

This study investigates the impact of design thinking pedagogy on college students’ career decision-making self-efficacy and perceived value in career education. A quasi-experimental design was employed with 140 participants from two homogeneous intact groups. The experimental group implemented design thinking pedagogy, whereas the control group received the regular teacher-centered method in career guidance courses. Data were collected using mixed methods. Quantitative data were collected using a career decision-making self-efficacy scale, and qualitative data were obtained through semi-structured interviews to explore students’ perceived value. Data were analyzed using independent samples *t* tests, analysis of covariance, and thematic analysis. The results revealed that design thinking pedagogy significantly improved students’ career decision-making self-efficacy through practical skill acquisition, emotional empowerment, and social identity, and perceived high evaluation, indicating that design thinking pedagogy is effective in career education. This study provides empirical evidence for integrating design thinking into career education, contributing to innovative pedagogies in higher education and emphasizing its importance in preparing students to proactively navigate evolving profession landscapes.

## Introduction

The rapid evolution of the job market demands more than academic knowledge from university students; it requires practical skills and career decision-making self-efficacy [1]. Career decision-making self-efficacy (CDMSE), as an individual’s confidence in performing tasks essential to career planning, is a critical predictor of career adaptability and employability [2]. In vocational psychology, CDMSE is a key factor in achieving sustainable career development [3]. With the growth of digitization and technology outpacing human adaptation, higher education is reevaluating instructional methods to meet the demands of the information era.

Career guidance courses, vocational skills training, and internships are essential components of college career development education [4]. In China, career guidance course are the most effective way to promote students’ career development, offering a scientific and systematic approach to teaching activities such as career, employment, and entrepreneurship education for students [5]. Third-year college students are typically enrolled in career guidance courses. However, most career guidance courses use a regular teacher-centered teaching approach that emphasizes theoretical learning and standardized content, neglects personal planning skills and practical learning, and limits college students career decision-making abilities [6].

Design thinking, developed at Stanford University, is a human-centered and iterative problem-solving methodology with universal applicability and multi-disciplinary [7,8]. It has the potential to improve classroom experience for both teachers and students and student learning [9]. As an innovative teaching method, design thinking emphasizes problem-solving, creativity, and teamwork, which are closely related to CDMSE [10]. The implementation of design thinking processes can help teachers create creative, interactive, engaging, and learner-centered classroom [11].

Design thinking pedagogy requires critical thinking, complex problem-solving, and critical reading skills [7], allowing students to express their perspectives through problem description, brainstorming, and group discussions and to ultimately find an inventive solution to a challenging problem [12,13]. The design thinking process based on the EDIPT model involves five steps: empathize(E), define(D), ideate(I), prototype(P) and test(T) [8]. Design thinking emphasizes empathy, prototyping, and user feedback [14], processes that closely align with the five dimensions of CDMSE: self-appraisal, career information gathering, goal selection, planning, and problem-solving [15]. Therefore, design thinking pedagogy can enhance the value and engagement of the curriculum for students and improve the quality of instruction. Aligned with a constructivist perspective on learning and development, design thinking pedagogy facilitates students’ experiential learning and focuses on the team-based learning process and iteration, thereby promoting the construction of knowledge and the development of problem solving skills and creativity [16]. It is goal-oriented and emphasizes problem-solving, teamwork, and dealing with uncertainty, all of which have the potential to increase students’ confidence in making career decisions.

Social Cognitive Career Theory is a research theory of social cognitive theory in the career field that provides a theoretical framework for career guidance to understand career behaviors and outcomes and develop career-related interventions. SCCT emphasizes the interactions between individuals, behaviors, and environments, and describes how people develop career interests, make career choices, and maintain career stability [17]. Self-efficacy, outcome expectations, career learning experience and personal-environmental factors are the core elements of SCCT [18]. SCCT-based interventions are useful for increasing students’ postsecondary awareness [19] and decision-making self-efficacy [20,21]. In SCCT-based career guidance, lecturers design learning environments and teaching activities (goal-oriented activities) to help students engage in learning situations, acquire career information search skills from problem-solving, and master job search skills. However, existing research on design thinking in education has predominantly focused on STEM fields [22] or entrepreneurship training [23], empirical research on design thinking pedagogy instruction in career guidance course seems to be insufficient and exploratory.

Thus, this study employed design thinking pedagogy in career guidance course, a quasi-experiment research design was conducted to examine the effect of design thinking pedagogy on students’ career decision-making self-efficacy and perceived value. The following research questions were investigated:

RQ1 Does design thinking pedagogy have a significant impact on undergraduate students’ CDMSE at a selected public university in Chaozhou City, Guangdong Province, China?

RQ2 What are students’ perceptions of design thinking pedagogy at a selected public university in Chaozhou City, Guangdong Province, China?

## Materials and methods

### Ethics approval and consent to participate

This study was approved by the Ethics Committee of Segi University (SEGiEC/SR/FOELPM/17/2023–2024). Additionally, the research was conducted with the consent of the dean of the relevant college. Before starting the study, participants provided written informed consent. The study was carried out from September 4 to November 3, 2023. Pre-test was conducted on 04/09/2023, while post-test was conducted on 30/10/2023. Students completed all questionnaires anonymously. Participants were also made aware that they had the right to withdraw from the study at any time without needing to give a reason. The researcher did not collect any confidential metadata from the participants. All data was securely stored on a computer protected by a unique password. After the experiment, students were informed about the nature of their treatment in both the experimental and control groups.

### Research site and participants

This study was conducted at a public university located in the eastern part of Guangdong Province, China. Within the education system in China, students choose their college majors based on their National College Entrance Examination scores. Then colleges and universities place students with the same major in one or more homogeneous classes. Students with the same major typically attend the same classes. In this study, approximately 5000 junior students in 17 faculties participated in career guidance courses.

Participants were 140 undergraduate students; all were third-year college students (20–21 years old) majoring in biological sciences who had to participate in career guidance course. The experimental group consisted of 70 students (male: 20, female: 50), while the control group consisted of 70 students (male: 22, female: 48). The required sample size for the independent samples *t* test comparing two parallel groups was determined using the G*Power Program1 Version 3.1.9.4. The analysis was based on a two-tailed test with an assumed medium effect size (Cohen’s d = 0.5), a significance level (α) of 0.05, and a desired power (1-β) of 0.90 [24]. The allocation ratio was set to 1, indicating equal group sizes. The results indicated that a minimum of 70 participants per group (140 total) were required to detect the specified effect size with the given parameters.

## Study design

This study examined the effects of design thinking pedagogy on college students’ career decision-making self-efficacy (CDMSE) and perceived value using a quasi-experimental, pretest–posttest design. A two-stage sampling method was employed to ensure sample representativeness. Firstly, stratified sampling was used to categorize all classes into four strata based on their academic major. Subsequently, one major was randomly selected from these strata using a random number generator. All intact classes (two classes) from the selected major were included in the study, with one class randomly assigned to the experimental group and the other to the control group. Due to administrative constraints within the university setting, random assignment of individual participants was not feasible. However, homogeneity of the two groups was ensured by selecting classes from the same major, academic year, and instructional background. The baseline CDMSE scores did not differ significantly between the groups (p > .05), confirming their equivalence prior to the intervention. One experienced instructor taught both classes to eliminate instructor effects, and both groups followed identical curricular modules.

In a 9-week experiment, students in the experimental group participated in weekly career guidance course with design thinking pedagogy, and students in the control group participated in career guidance classes with a regular teacher-centered teaching method. The effects of design thinking intervention (RQ1) on CDMSE were determined through the pretest and posttest.

Participants in the qualitative phase were selected using purposive sampling [25] for semi-structured interviews to explore their perceptions toward design thinking pedagogy (RQ2). The experimental group was divided into eight groups for learning, and interviewers were selected from each group to understand their perceptions of the design thinking pedagogy. Each group leader then completed a semi-structured interview with the researcher to provide rich perceptual data for triangulation with the quantitative findings. The eight students interviewed were identified as S1, S2, S3...., S8.

### Educational intervention

**Fig 1:**
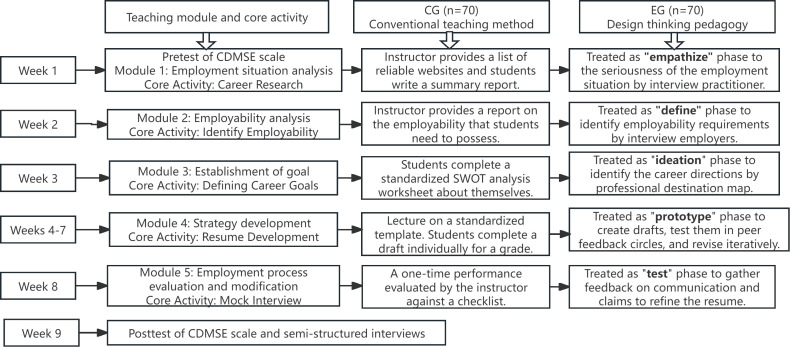
The intervention procedure. *Note.* SWOT: S – Strengths; W – Weaknesses; O – Opportunities; T – Threats; CG – control group; EG – experimental group; CDMSE – career decision-making self-efficacy.

According to the Teaching Requirements for College Student Career Guidance Course issued by the Office of the Ministry of Education in China, the career guidance course guides students to master job search skills through self-analysis, environmental analysis, understanding the employment situation, and clarifying the employment skills that meet the requirements of employers. The career guidance course was taught for a total of 9 weeks and 18 credit hours and consisted of five modules: employment situation analysis, employability analysis, goal establishment, strategy development, and employment process evaluation and modification [26]. Both groups received identical curricular content; however, their pedagogical approaches differed fundamentally, as illustrated in Fig 1.

During the first week, both the experimental and control groups completed the pretest, including the CDMSE scale. The experimental group was taught using design thinking pedagogy, while the control group using the conventional teaching method. In the control group, the regular teacher-centered teaching method entailed a lecturer delivering information, while students passively received and pondered to obtain knowledge and abilities. In the experimental group, the lecturer implemented design thinking pedagogy into the career guidance course. In Week 9, both the experimental and control groups completed the posttest, including the CDMSE scale. Ten students were selected for semi-structured interviews.

For the experimental group, the five-step EDIPT model from the Stanford University School of Design, as defined in the Introduction, was applied. Each stage was systematically mapped to specific career guidance competencies through carefully designed learning activities. The empathize stage (week 1), corresponding to the “Employment situation analysis and employability analysis” module, where students conducted a personal SWOT analysis and stakeholder interviews using a semi-structured protocol to deeply understand career challenges, perspectives and employer needs, culminating in an “Empathy Map” Activities included empathy mapping and persona development to enhance understanding of career contexts. Based on empathy work, the define stage (week 2) for “Establishment of goal”, where students involved synthesizing insights into actionable problem statements. Students identify employability requirements by interview employers. During the ideate stage (week 3), participants engaged in structured brainstorming techniques, such as mind mapping, and SCAMPER techniques to produce diverse career solutions. Each student developed at least three alternative career pathways with corresponding action plans. In the prototype stage (week 4–7) with the strategy development module, students transformed ideas into tangible forms, such as creating draft resumes, cover letters, and digital portfolios. They developed 30-second self introduction and practiced them in small groups. Finally, the test stage (week 8) for employment process evaluation and modification module, where students participated in simulated job interviews, received structured peer feedback, and iteratively refined their prototypes based on the insights gained, resulting in a finalized portfolio of career application materials and a reflection on the iterative improvement process.

**Fig 2:**
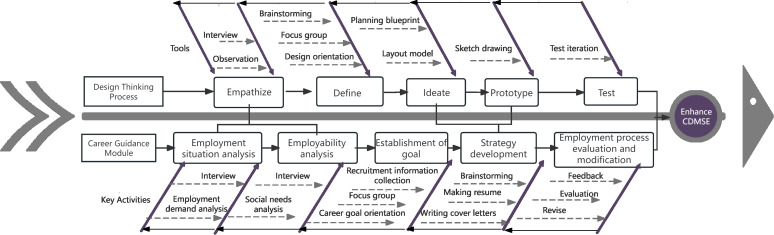
Framework for integrating design thinking pedagogy into career guidance. *Note.* CDMSE – career decision-making self-efficacy.

The career guidance curriculum encompassed five modules aligned with the five stages of design thinking. Fig 2 introduces a framework that integrates the EDIPT model with the five core modules of the career guidance course adapted from Lin and Wang (2022). Styled as an Ishikawa diagram, the figure positions the iterative design thinking process along its central spine, driving the desired outcome, and enhancing CDMSE. Each lateral bone represents one phase and explicitly links that phase to the corresponding curricular module and the targeted pedagogical tools (e.g., stakeholder interviews during the empathize phase). In terms of practice, each group selected a practical task to apply the steps and tools of design thinking, and the resulting design was shared and evaluated to determine the direction of career guidance. This visualization demonstrates that design thinking functions not as an ancillary activity, but as a primary student-centered engine guiding the entire course.

### Instruments

#### Career decision-making self-efficacy scale.

The CDMSE scale was adapted from Peng and Long [27], which was adapted from Betz and Taylor’s CDMSE scale for China. The scale includes five dimensions: self-evaluation (SA; six items), information gathering (IG: nine items), goal selection (GS: nine items), making a plan (MP: eight items) and problem-solving (PS: seven items), with a total of 39 items using a 5-point Likert scale from 1 (*fully not confident*) to 5 (*fully confident*). In a prior pilot study conducted with a sample from the same university and discipline [28], the scale demonstrated strong psychometric properties. Confirmatory factor analysis (CFA) confirmed the scale’s factorial structure, with high internal consistency (Cronbach’s alpha = 0.862) and good convergent validity (average variance extracted, AVE = 0.783). For the present study, the scale was administered without modifications. Internal consistency was re-examined using the current sample’s pretest data (N = 140). The Cronbach’s alpha coefficient for the scores from CDMSE scale was 0.945, indicating high reliability. The average variance extracted (AVE) value was 0.783, indicating good convergent validity of the variables.

***Semi-structure interviews*:** In addition to the quantitative data collection and analysis, this study conducted semi-structured interviews with eight students from the experimental group to understand their perceived attitudes towards design thinking pedagogy.

The interview outline presented in Table 1 was constructed by Yan [29,30] along with feedback from other lecturers with experience teaching career guidance courses. RQ 2.1 identifies the most influential design thinking activities, and RQ 2.2 links these experiences to changes in self-efficacy (CDMSE). Given that design thinking is an inherently collaborative pedagogy that emphasizes teamwork, peer feedback, and co-creation, the process of receiving input and having one’s ideas validated by peers serves as a critical form of social persuasion—a key source of self-efficacy in Bandura’s theory, underlying SCCT (Lent & Brown, 2019). Thus, RQ2.3 examines whether these social interactions, central to design thinking, contribute to enhancing students’ confidence in making career decisions.

**Table 1:** Interview outline.

Number	Questions
RQ 2 What are students’ perceptions of design thinking pedagogy in a selected public university in Chaozhou City, Guangdong Province, China??	
RQ2.1	Which experiences in the course have had the greatest impact on your career decision-making ability?
RQ2.2	How have these experiences changed your confidence in your career abilities?
RQ2.3	Do you feel recognized by others, such as teachers and classmates? How does this recognition affect you?

### Data collection and analysis

The process for data collection and analysis adopted a mixed method to better address the research questions and obtain more in-depth and accurate results. After obtaining permission from the deans of the identified faculty members to conduct the study, quantitative data were collected using a CDMSE scale in the pretest and posttest using an online survey. During these tests, if a respondent missed or skipped a question, the survey tool reminded them of the missed question and allowed them to answer it before submitting it to reduce missing questionnaire data. Thus, no data were missing in the quantitative data collection.

Qualitative data were collected through in-depth semi-structured interviews after the intervention. In the experimental group, all participants were divided into 8 groups for group discussions during the course. The leader of each group was selected to be interviewed by the researcher, and a total of 8 students were interviewed; interviews ranged from 35 min to 1 h and were conducted in Chinese and audiotaped. The researcher asked the interview questions listed in Table 1; each participant answered the same questions. The interviewer asked additional questions based on their responses and encouraged the interviewees to share their input on aspects that the interviewers had not requested in order to provide suggestions for conducting the course later.

Quantitative data were processed through descriptive statistic, normality of distribution, inferential statistics (including independent samples *t* tests and paired sample *t* tests), and analysis of covariance (ANCOVA) in SPSS 25.0, while qualitative data were analyzed through thematic analysis to synthesize both types of data to verify the effects of design thinking on the CDMSE and perceived value.

First, a normal distribution test was conducted to determine whether the sample data followed a normal distribution- a prerequisite for parametric statistical analysis. Normality of distribution was performed in SPSS 25.0 by the Kolmogorov-Smirnov test in this study. The significant values of the pretest and posttest of the experimental group and control group for CDMSE (experimental group: *p* = .200, *p* = .160, respectively; control group: *p* = .200, *p* = .200, respectively) were all above.05, indicating that the data follows a normal distribution.

Inferential statistics, such as the independent samples *t* test, were used to analyze the differences in scores between the experimental and control groups on the pretest and posttest. Cohen’s d was used to describe the effect size of the independent sample *t* test. Using pretest scores as a covariate to account for initial pretest differences, an ANCOVA was performed to examine the differences between the two groups’ posttest scores. All assumptions for parametric tests were verified, including normality (Kolmogorov-Smirnov tests, p > .05) and homogeneity of variance (Levene’s test, p > .05).

Semi-structure interview data were analyzed using thematic analysis following the framework proposed by Braun and Clarke [31]. The interview results were audio-recorded and transcribed sentence by sentence. Based on the transcribed text, two researchers independently coded the first three transcripts to identify salient features and concepts relevant to the research questions. A preliminary codebook was developed from this process. Then, online-by-line coding and thematic analysis were performed, which entailed highlighting key sentences that showed participants’ situations, actions, and processes. The potential themes were reviewed and refined in relation to the coded extracts and the entire dataset. Furthermore, the essence of each theme was articulated and clear definitions were established. In this study, the researcher transcribed audio data from interviews with learners into textual data to categorize document data on the eight students’ perceptions of design thinking pedagogy in terms of their ability development, cognitive development, and affective attitudes. The documents were coded according to the three areas, and key ideas from the process were labeled for thematic analysis to answer RQ2.

To ensure the reliability and consistency of the coding process, inter-coder reliability was formally assessed. The two researchers independently coded all eight transcripts using the finalized codebook. The degree of agreement was calculated using Cohen’s Kappa coefficient, which yielded a score of κ = 0.75, indicating a substantial level of agreement between coders. All discrepancies were thoroughly discussed until a full consensus was reached for final code application, thereby ensuring the credibility and consistency of the thematic structure.

## Results

### The effect of design thinking on participants’ self-efficacy scores in professional decision-making

Table 2 shows the means of students’ CDMSE scores in the experimental and control groups. The posttest CDMSE scores of the experimental group (*M* = 3.83, *SD* = 0.35) were significantly higher than those of the control group (*M* = 3.49, *SD* = 0.51). The improvement in the CDMSE scores of the experimental group after the intervention indicates that design thinking pedagogy may have a positive impact on CDMSE.

**Table 2:** Career decision-making self-efficacy scores of the participants in the experimental and control groups before and after the intervention.

Variables	Group	Control group(n = 70)	Experimental group(n=70)	Independent sample t-test	P-value	Cohen’s d
SA	Pre-test (X ± SD)	3.20 ± 0.70	3.24 ± 0.62	−0.38	0.703	–
	Post-test(X ± SD)	3.38 ± 0.70	3.76 ± 0.51	−3.65	0.000	0.62
IG	Pre-test (X ± SD)	3.31 ± 0.54	3.30 ± 0.45	0.11	0.910	–
	Post-test(X ± SD)	3.47 ± 0.56	3.82 ± 0.42	−4.16	0.000	0.71
GS	Pre-test (X ± SD)	3.35 ± 0.54	3.33 ± 0.51	0.25	0.802	–
	Post-test(X ± SD)	3.52 ± 0.54	3.84 ± 0.49	−3.73	0.000	0.62
MP	Pre-test (X ± SD)	3.41 ± 0.54	3.47 ± 0.52	−0.66	0.509	–
	Post-test(X ± SD)	3.56 ± 0.57	3.87 ± 0.44	−3.61	0.000	0.61
PS	Pre-test (X ± SD)	3.26 ± 0.57	3.22 ± 0.50	0.43	0.670	–
	Post-test(X ± SD)	3.51 ± 0.52	3.84 ± 0.49	−3.86	0.000	0.65
CDMSE	Pre-test (X ± SD)	3.31 ± 0.49	3.31 ± 0.45	−0.08	0.937	–
	Post-test(X ± SD)	3.49 ± 0.51	3.83 ± 0.35	−4.58	0.000	0.78

SA: self-evaluation; IG: information gathering; GS: goal selection; MP: making plan; PS: problem solving; CDMSE: career decision-making self-efficacy; SD = standard deviation.

There was no significance difference between the experimental and control groups regarding students’ pretest self-evaluation, information gathering, goal selection, making a plan, problem solving, and CDMSE scores (Table 2). However, after the intervention, a significant difference was found in the students’ CDMSE scores between the control group (*M* = 3.49, *SD* = 0.51) and the experimental group (*M* = 3.83, *SD* = 0.35; *t* (138) = −4.58, p < .001, two-tailed). Additionally, a moderate significant difference was found in the total mean scores of CDMSE between the groups (Cohen’s *d* = 0.78; Table 2).

A one-way ANCOVA was conducted with pretest CDMSE scores entered as covariates. The ANCOVA revealed a significant between-group difference on posttest scores, *F* (1,137) = 20.86, *p* < .001, η^2^ₚ = .132 (Table 3). The covariate itself did not exert a significant effect (*p* > .05, η^2^ₚ = .001). Thus, the career guidance course with design thinking pedagogy significantly enhanced students’ CDMSE. This improvement is primarily attributable to interventions employing design thinking in career guidance courses.

**Table 3:** Analysis of covariance results of career decision-making self-efficacy on the posttest.

Group	*N*	Mean	SD	*F*	*p* value	*η*^2^(group)	*η*^2^(pretest)
Experimental group	70	3.83	0.352	20.858	.000	.132	.001
Control group	70	3.49	0.507				

### Students’ perceptions of design thinking

To investigate the participants’ perceptions of design thinking pedagogy, this study interviewed eight students from the experimental group. Based on the interview transcription results, seven conceptual codes were extracted and grouped into three themes (Table 4): practical skills, emotional empowerment, and social identity.

**Table 4:** Themes emerging from students’ perceptions of design thinking.

Themes	Conceptual codes	Interviewees	Number
Practical skills	Problem-solving ability	S1, S2, S4, S5, S6, S7	6
	Innovation ability	S2, S4, S5, S7, S8	5
	Teamwork ability	S1, S3, S4, S5, S7, S8,	6
Emotional empowerment	Increased self-confidence	S2, S4, S5, S7, S8	5
	Enhanced learning motivation	S1, S2, S3, S4, S6, S8	6
Social identity	Classmate recognition	S1, S2, S3, S4, S5, S6, S7, S8	8
	Teacher feedback	S1, S2, S3, S4, S5, S6, S7, S8	8

Regarding practical skills, students generally believed that design thinking pedagogy provides practical skills directly related to career development. Eight students believed that they had mastered their problem-solving skills. One of the students (S1) said, “*The projects in the course require us to solve real career problems, such as simulated recruitment. This taught me how to break down complex problems and find feasible solutions.*” Another student (S6) said, *“We didn’t just write a resume and turn it in. We went through multiple rounds—first with classmates, then with our mentor. Each time, I found something new to improve, from the wording of a bullet point to how I presented my projects. It taught me that solving a problem like ‘how to market myself’ isn’t about getting it perfect the first time, but about being willing to test and improve.”*Five students felt that design thinking pedagogy improved their innovation skills. One student (S4) said, “*I always felt that I lacked creativity before, but the ‘brainstorming’ part of design thinking made me realize that innovation can be trained. I can have many career choices before I make career decisions.*” Six students believed that they had gained teamwork skills: “*In the group project, I learned how to cooperate with classmates. This experience made me more confident in future teamwork*” (S8).

Participants’ responses showed that the design thinking process can emotionally empower students. Students experienced significant emotional changes throughout the course that were closely related to their CDMSE. Five students felt greater self-confidence: “*The process of multiple iterations of prototypes taught me that after completing my resume, I found that I could actually solve seemingly impossible tasks. This sense of achievement made me less anxious about my career choice*” (S7). Six students believed their learning motivation was enhanced. “*The course content is close to reality and not as boring as regular classes. I am now more willing to actively explore career information*” (S8). In addition, one student (S1) said that ‘*I enjoy this type of class because it encourages participation, helps me learn to put myself in the position of the employer by considering the qualifications and requirements I must meet, and then challenges me to keep improving. For instance, learn how to gather employment-related information, comprehend the professional employment destination, and decide on career goals before creating a resume and getting ready for job applications, etc.’*

Design thinking activities can provide social support. The social recognition that the students gained during the course further strengthened their confidence in their career decision-making. All students felt that they had received recognition from their classmates and feedback from the instructor during the course: *“When my solution was adopted by group members, I felt that my value was recognized, which made me more confident in my career judgme*nt” (S6). *“The teacher’s detailed feedback on our resumes and mock interviews made me realize that my career planning is feasible*” (S4).

## Discussion

This study provides empirical support for the integration of design thinking pedagogy into SCCT-based career guidance, demonstrating the positive effect on students’ career decision-making self-efficacy (CDMSE). Quantitative results confirmed significant improvements in all CDMSE dimensions with moderate effect sizes, while qualitative findings elucidated the underlying mechanisms, offering a coherent explanation for the improvement.

Specifically, during the define phase of design thinking, students are encouraged to reflect on their skills and preferences through activities like self-portraits or personal storytelling. This helps them achieve more accurate self-assessment, which is crucial to CDMSE. During the ideate phase, students are exposed to a wide range of career possibilities and comprehend the current employment landscape. They research different industries and job roles, which broadens their career horizons and leads to a significant increase in their CDMSE gathering-information scores. The significant improvement in self-evaluation scores (d = 0.61) corresponds with students’ reports of gaining clearer self-awareness through stakeholder interactions. Furthermore, through the core stages of design thinking, prototyping and testing, students develop resumes, conduct mock interviews, and continuously refine their solutions based on teacher feedback. This cyclical process fosters a growth-oriented mindset by normalizing failure as a step toward improvement [32]. This aligns with Bandura’s theory of mastery experiences [33], as iterative testing enables students to achieve incremental successes, thereby enhancing their confidence in managing complex career choices. As one student noted: ‘Each resume revision based on feedback made me more confident in my ability to present myself professionally.’ This direct experience with career development tasks aligns with Bandura’s emphasis on enactive mastery as the most powerful source of self-efficacy beliefs. In addition, the results indicate that design thinking equips students with problem-solving skills that are directly applicable to career decision-making. They learned to identify career-related problems, generate multiple solutions, and evaluate the best course of action. These findings are consistent with those of other studies [12,16,34] that reported design thinking can enhance students’ problem-solving skills. In the SCCT framework, the ability to handle career-related challenges boosts self-efficacy.

From the perspective of the student leaders interviewed, qualitative analysis identified three main ways in which design thinking pedagogy promoted CDMSE, corroborating the significant rise in CDMSE scores observed in the experimental group within quantitative findings. These participants reported directly enhance their self-efficacy in coping with career challenges by mastering practical skills such as problem-solving, innovation, and teamwork. This explains the cognitive basis for the experimental group’s increase in quantitative CDMSE scores. Design thinking pedagogy creates a learning environment that makes students feel more at ease when confronted with obstacles. Tasks may be completed by working in groups, which helps students enhance their collaboration and communication skills. Integrating design thinking into the curriculum dramatically increases students’ creativity and self-assurance [33,35,36]. Additionally, emotional trajectories such as emotional empowerment (including self-confidence) reduce career decision-making anxiety and indirectly boost self-efficacy. Design thinking was found to trigger greater intrinsic motivation in students; this innovative teaching method can stimulate their interest in learning and enhance their willingness to explore the professional world. Students experience more enjoyment when designing resumes and cover letters. The feelings during creative activities are closely related to internal motivation, and the generation of intrinsic motivation is a source of personal creativity and sense of efficacy [36]. Furthermore, it is socially driven. In the design thinking process, the emphasis on improving communication and collaboration skills correlates with the core principles of a constructivist learning approach [37]. The collaborative nature of design thinking also provided social persuasion and vicarious learning opportunities—additional key sources of self-efficacy in SCCT. Students reported learning from observing peers’ approaches to career challenges and receiving constructive feedback throughout the process. External reinforcement from peer and teacher acknowledgment helps individuals develop lasting self-efficacy beliefs [18]. This supplements the social context factors that were not assessed in this quantitative study.

Our findings validate the cognitive advantages of design thinking within the context of career education. Previous research has shown the advantages of fostering critical thinking by prompting students to recontextualize challenges [38,39]. Researchers focused on generic problem-solving skills; however, our study connects these talents to career decision-making by demonstrating how iterative prototyping transforms abstract self-reflection into effective tactics. This specificity addresses a gap in career education, which often prioritizes theory over experiential skill-building [6]. In contrast to other studies that focused solely on the influence of design thinking on creativity [40–42], our CDMSE scales measured psychological benefits and demonstrated enhancements in self-assessment and goal setting, which are crucial elements of career readiness.

## Conclusion

This study demonstrates that employing design thinking pedagogy in career guidance can significantly improve college students’ CDMSE and perceived value. Quantitative analysis confirmed a statistically significant increase in CDMSE scores, and qualitative results revealed three enhancing pathways: practical skill acquisition, emotional empowerment, and social identity.

This study addresses the lack of empirical research on design thinking in career education and provides an innovative, practical path for enhancing students’ career preparedness within similar disciplinary and institutional contexts. Furthermore, it contributes to extending the application of design thinking to the domain of undergraduate career development, thereby enriching the growing discourse on innovative pedagogies in 21st-century career education.

## Implications

This study offers preliminary evidence for integrating design thinking into college career guidance within a specific institutional and disciplinary context. The core concepts of design thinking, such as human-centered and iterative innovation, can be combined with SCCT to provide a new theoretical perspective for career intervention. For example, the prototyping and testing phases of design thinking can be used as an action-oriented self-efficacy enhancement method to improve student CDMSE, thereby overcoming the shortcomings of conventional teacher-centered teaching methods.

In addition, within the scope of this study, the five steps of design thinking can be applied to career guidance course activities, such as incorporating real career problems (e.g., mock interviews) into career development courses and guiding students through these five processes to solve problems. When effectively implemented, these elements of design thinking can affect students’ career objective selection, problem-solving, career information gathering, and job search skills, all of which are crucial for their long-term career development. Career-planning teachers in comparable contexts may consider collaborating with experts in fields such as design and psychology to develop program content and assessment tools.

Furthermore, at the institutional level, in career counseling, design thinking tools such as user journey mapping and stakeholder analysis can be used to help students visualize contradictions and opportunities in career choices. For example, by drawing a user journey map of career roles, students can more intuitively identify the potential challenges and opportunities of different career occupations. Qualitative results showed that emotional empowerment is a key driver of career decision-making and that emotional management can be designed in counseling to help students cope with decision-making anxiety. Given the exploratory nature of these findings and the single‑institution, single‑discipline sample, further research is needed to examine their transferability to other educational contexts.

## Strengths and limitations

This study systematically integrates design thinking to college career guidance. It overcomes the shortcomings of regularly teacher-centered teaching method by demonstrating how experiential learning can increase students’ CDMSE using the iterative, human-centered framework of design thinking, particularly prototyping and testing. To ensure reliability and validity, quantitative data from the CDMSE scale and qualitative data from students’ subjective experiences and opinions are utilized to assess the effectiveness of the design thinking pedagogy. This study also addresses the lack of design thinking in career education, which improves students’ career preparedness and policymaking.

However, there are several limitations that warrant consideration. The main limitation of this study is the generalizability of the sample. Due to practical constraints, this study did not provide a comprehensive review of all undergraduate students; thus, the study results lack generalizability due to the single-institution, single-discipline sample bias. Future studies should consider increasing the sample size across different institutions and disciplines to further validate the generalizability of the results. Additionally, the qualitative component was based solely on interviews with group leaders, which may limit the diversity of perspectives captured. Future research should include voices from a broader range of participants, including non-leaders, to obtain a more comprehensive understanding of student experiences. The quasi-experimental design with intact groups, though practical in educational settings, limits strong causal claims. Although we established pre-test equivalence and used ANCOVA to control for baseline scores, unmeasured confounding variables may influence results. The moderate-to-large effect sizes observed (Cohen’s d ranging from 0.61 to 0.78) may reflect both the intervention’s efficacy and the novelty effect of an innovative pedagogy. Future research should employ randomized controlled trials across multiple institutions to enhance generalizability. Additionally, the absence of long-term follow-up limits understanding of sustained effects; future studies should include 6-month and 12-month assessments of career outcomes and adaptability.

## Supporting information

S1 File Data set files. (XLSX)
